# To: The Perme scale score as a predictor of functional status and complications after discharge from the intensive care unit in patients undergoing liver transplantation

**DOI:** 10.5935/0103-507X.20190082

**Published:** 2019

**Authors:** Márcio Ferreira Camillis, Lucas Galant, Leonardo Miguel Correa Garcia, Ricardo Wickert, Priscila Cidade, Cassiano Teixeira

**Affiliations:** 1 Serviço de Fisioterapia, Hospital Moinhos de Vento - Porto Alegre (RS), Brasil.; 2 Serviço de Hepatologia, Universidade Federal de Ciências da Saúde de Porto Alegre - Porto Alegre (RS), Brasil.; 3 Unidade de Terapia Intensiva, Hospital Moinhos de Vento - Porto Alegre (RS), Brasil.

**To the Editor**

I would like to congratulate the authors responsible for the article entitled "The Perme scale score as a predictor of functional status and complications after discharge from the intensive care unit in patients undergoing liver transplantation", the topic of which is extremely relevant to the academic community.^(1)^

This topic, addressing the functional assessment of this patient population, which is highly affected by loss of muscle strength and functionality during the course of the disease and at times of hospital admission, is extremely relevant. For this reason, it raised much interest, and we would like to add some suggestions and observations that we believe are relevant to the study.

Patients with cirrhosis who are candidates for transplantation present with important muscle dysfunction over the course of the disease. Such dysfunction includes changes in muscle mass and strength, which are often associated with changes in functional fitness - factors that limit quality of life and increase mortality before transplantation.^(2)^

One aspect related to the worsening of functional status is the time spent on the liver transplant waiting list. In the article, there was no mention of controlling for this variable, which may influence the main outcome of the study. Another criterion to be mentioned is the participation of patients with cirrhosis and hepatopulmonary syndrome, a condition that may be present in up to 30% of patients with cirrhosis. The authors did not exclude this patient subgroup, which may be present in the respective sample and may influence aspects related to dyspnea.^(3)^

Regarding the instrument used to measure patient functional status, we suggest the use of another tool because the Perme Intensive Care Unit Mobility Score is a scale developed specifically to evaluate patients admitted to the intensive care unit, and it cannot be used in settings such as inpatient units or outpatient clinics.^(4)^
